# The Impact of Bisphenol A (BPA) on Serotonin Regulation and Its Implications for Mood

**DOI:** 10.1192/j.eurpsy.2025.1534

**Published:** 2025-08-26

**Authors:** R. A. Maldonado-Puebla, N. Choudhury, B. Carr

**Affiliations:** 1 College of Osteopathic Medicine, Nova Southeastern University, Clearwater; 2 Psychiatry, University of Florida, Gainesville, United States

## Abstract

**Introduction:**

Bisphenol A (BPA), a widely used chemical in the manufacturing of plastics and resins found in everyday consumer products such as water bottles and food containers, is known to interfere with the body’s endocrine system. Specifically, BPA acts as a xenoestrogen, meaning it can mimic the hormone estrogen by binding to estrogen receptors (ERs) in the body. This interaction raises concerns about BPA’s potential impact on brain regions with high densities of these receptors, including the hippocampus and prefrontal cortex, which are critical for mood and cognitive functions. Estrogen plays a vital role in regulating serotonin, a neurotransmitter essential for mood stability. This review synthesizes existing literature on how BPA may disrupt serotonin regulation and its implications for mood disorders.

**Objectives:**

To evaluate the mechanisms by which BPA interacts with estrogen receptors and serotonin in the brain with a key focus on its implications for mood disorders such as depression and anxiety.

**Methods:**

A narrative literature review was conducted, gathering findings from relevant studies published over the past two decades. Sources were identified through database searches in PubMed, PsycINFO, and Google Scholar using keywords such as “BPA,” “serotonin,” “estrogen receptors,” “hippocampus,” “prefrontal cortex,” and “mood disorders.” The review focused on various types of studies that examine BPA’s interaction with estrogen receptors and its effects on serotonin regulation allowing for the creation of an up-to-date integrative summary.

**Results:**

The review reveals that BPA’s interaction with estrogen receptors in the brain can lead to significant disruptions in serotonin regulation. BPA competes with endogenous estrogen for ER binding leading to altered serotonin synthesis and release. BPA exposure is associated with reduced expression of tryptophan hydroxylase, the enzyme critical for serotonin production, potentially lowering serotonin levels. BPA’s interference with ERs impairs serotonin release from presynaptic neurons, particularly in the hippocampus and prefrontal cortex, which are vital for mood regulation.

**Conclusions:**

The literature strongly suggests that BPA disrupts normal estrogenic regulation of serotonin, with significant implications for mood disorders such as depression and anxiety. The hippocampus and prefrontal cortex appear particularly vulnerable to these disruptions, which could theoretically exacerbate symptoms of mood dysregulation highlighting the importance of considering environmental factors like BPA in the treatment of mood disorders. Given the widespread exposure to BPA, these findings underscore the need for further research into its long-term effects on mental health and potential regulatory measures to mitigate exposure.

**Disclosure of Interest:**

None Declared

